# Improvement of Cardiac Function in Mouse Myocardial Infarction after Transplantation of Epigenetically-Modified Bone Marrow Progenitor Cells

**DOI:** 10.1371/journal.pone.0022550

**Published:** 2011-07-22

**Authors:** Johnson Rajasingh, Jayakumar Thangavel, Mohammad R. Siddiqui, Ignatius Gomes, Xiao-pei Gao, Raj Kishore, Asrar B. Malik

**Affiliations:** 1 Department of Pharmacology and Center of Lung and Vascular Biology, University of Illinois College of Medicine, Chicago, Illinois, United States of America; 2 Feinberg Cardiovascular Research Institute, Northwestern University School of Medicine, Chicago, Illinois, United States of America; University of Illinois at Chicago, United States of America

## Abstract

**Objective:**

To study usefulness of bone marrow progenitor cells (BPCs) epigenetically altered by chromatin modifying agents in mediating heart repair after myocardial infarction in mice.

**Methods and Results:**

We tested the therapeutic efficacy of bone marrow progenitor cells treated with the clinically-used chromatin modifying agents Trichostatin A (TSA, histone deacetylase inhibitor) and 5Aza-2-deoxycytidine (Aza, DNA methylation inhibitor) in a mouse model of acute myocardial infarction (AMI). Treatment of BPCs with Aza and TSA induced expression of pluripotent genes Oct4, Nanog, Sox2, and thereafter culturing these cells in defined cardiac myocyte-conditioned medium resulted in their differentiation into cardiomyocyte progenitors and subsequently into cardiac myocytes. Their transition was deduced by expression of repertoire of markers: Nkx2.5, GATA4, cardiotroponin T, cardiotroponin I, α-sarcomeric actinin, Mef2c and MHC-α. We observed that the modified BPCs had greater AceH3K9 expression and reduced histone deacetylase1 (HDAC1) and lysine-specific demethylase1 (LSD1) expression compared to untreated BPCs, characteristic of epigenetic changes. Intra-myocardial injection of modified BPCs after AMI in mice significantly improved left ventricular function. These changes were ascribed to differentiation of the injected cells into cardiomyocytes and endothelial cells.

**Conclusion:**

Treatment of BPCs with Aza and TSA converts BPCs into multipotent cells, which can then be differentiated into myocyte progenitors. Transplantation of these modified progenitor cells into infarcted mouse hearts improved left ventricular function secondary to differentiation of cells in the niche into myocytes and endothelial cells.

## Introduction

Bone marrow-derived progenitor cells (BPCs) and endothelial progenitor cells (EPCs) from the bone marrow can mediate neovascularization in the ischemic myocardium and improve pump function of the heart [1], [2]. However, stem cell therapy involving BPCs and EPCs has often produced small and variable effects and, importantly, it remains unclear whether these cells are capable of inducing myogenesis and myocardial regeneration [3], [4]. The protective effects also appear to be due to release of humoral factors [5]. Because of the variability of the responses using BPCs and EPCs [3]–[5], we addressed the question whether BPCs can be improved by modifying them pharmacologically such that there is improved engraftment and myocardial regeneration. Here we used the strategy of treating BPCs with available chromatin modifying agents prior to their transplantation. We used trichostatin A (TSA, a histone deacetylase inhibitor) and 5Aza-2-deoxycytidine (Aza, a DNA methylation inhibitor) [6] based on the precept that DNA demethylation and histone acetylation are key steps required for epigenetic modification of cells [7], [8]. These agents have been shown to alter cell fate [9], [10] and differentiate mesenchymal stem cells (MSCs) into cardiac myocytes (CMCs) [11]. We observed that these drugs used together induced conversion of BPCs to multipotent cells (referred to as eiBPCs). The cardiac progenitor cells generated from eiBPCs were used for transplantation into mouse infarcted hearts. We investigated how these agents converted BPCs into multipotent eiBPCs and effects of transplantation of cardiac progenitors derived from eiBPCs on cardiac function. We observed that combined Aza and TSA treatment of BPCs induced epigenetic changes characterized by greater AceH3K9 expression and reduced histone deacetylase1 (HDAC1) and lysine-specific demethylase 1 (LSD1) expression compared to untreated BPCs. These epigenetic changes induced expression of Oct4, Nanog and Sox2, transcription factors in BPCs. Transplantation of cardiac progenitors derived from eiBPCs into infarcted mouse hearts significantly improved left ventricular function that was coupled to differentiation of the injected cells into CMCs and endothelial cells at sites of transplantation.

## Materials and Methods

### Cell culture

Mouse bone marrow derived progenitor cell (BPC) culture was carried out as described [12]. Mononuclear cells isolated from the tibias and femurs of GFP-expressing transgenic C57BL/6-TgN (ACTbEGFP) mice were cultured in EBM-2 medium supplemented with (SingleQuot Kit; Clonetics) 5% FBS on cell-culture dishes coated with 0.1% rat vitronectin/gelatin. After 4 days in culture, the adherent cells were reseeded (5×10^4^ cells/cm^2^) on 0.1% vitronectin/gelatin-coated 10 cm culture dishes and 4-well chamber slides and cultured for 3 additional days. Cells in 4-well slides were co-incubated with DiI-acLDL (Biomedical Technologies) for 1 hour, stained with fluorescein isothiocyanate–conjugated *Banderia simplicifolia* lectin 1 (FITC-BS1), and viewed under fluorescence microscope. Majority of these cells were double-stained with these markers defining them as endothelial-lineage cells [12]. These cells were further characterized by flow cytometry using stem and progenitor cell marker antibodies. Day-7 BPCs growing in 10 cm culture dishes were used for the chemical modification experiments using Aza and TSA described below.

### Quantitative reverse transcriptase-polymerase chain reaction (qRT-PCR)

Day-7 BPCs were treated with Aza (0, 10, 25, 50 and 100 nM) or TSA (0, 5, 10, 25 and 50 nM) or both drugs combined for 4 days. Total cellular RNA was obtained for qRT-PCR analysis to determine mRNA expression of pluripotency markers [13], [14]. The gene expression profiles of Primer and probe sequences specific for specific target genes are given in Table 1. Relative mRNA expression of target genes was normalized to endogenous 18S control gene (Applied Biosystems). Results were expressed as fold change in expression and values were calculated as ratio of induced expression-to-control expression.

**Table 1:** Primers and Sequences.

Primers used for qRT-PCR	
Gene name	Sequences
CTT	F: GACAGACTGGTCGGGAGATGA R: CCCCCATGTAGTCGATGTTCA
C-Kit	F: TGGCTCTGGACCTG R: ACAATTCTTGGAGGCGAGGAA
eNos	F: TCTGCGGCGATGTCACTATG R: CATGCCGCCCTCTGTTG
Gata4	F: GCGCCCCATCAAGACAGA R: GTGGCCGGACACAGTACTGA
Nanog	F: GCTCAGCACCAGTGGAGTATCC R: TCCAGATGCGTTCACCAGATAG
Nkx2.5	F: CTTCAAGCAACAGCGGTACCT R: CGCTGTCGCTTGCACT
Oct4	F: TCGGACCAGGCTCAGAGGTA R: ATCCCTCCGCAGAACTCGTA
Sox2	F: AAACCAAGACGCTCATGAAGAAG R: CGCTCGCCATGCTGTTC
VE-Cadherin	F: GTGGATGAGCCCCCTGTCT R: CAGCGGTTTCTTC
Flk-1	F: GAGCTCTCCGTGGATCTGAAA R: AACAAAGCCTGAG
IL-10	F: CAGCCGGGAAGACAATAACTG R: CCGCAGCTCTAGGAGCATGT
IL-10R	F: TGTCTGTATGCAAAGCTTGGAAAT R: GTCTGTGCCCGCTTTCTCA
TNF-α	F: GGCTGCCCCGACTACGT R: AGGTTGACTTTCTCCTGGTATGAGA
MCP1	F: CTTCCTCCACCACCATGCA R: CCAGCCGGC
IL-6	F: TTCCATCCAGTTGCCTTCTTG R: GGGAGTGGTATCCTCTGTGAAGTC
IL-1β	F: CTACAGGCTCCGAGATGAACAAC R: TCCATTGAGGTGGAGAGCTTTC
**Primers used for PCR**	
**eGFP** (315 bp):	F: 5′- ATG GTG AGC AAG GGC GAG GAG CTG -3′ R: 5′- GCC GTC GTC CTT GAA GAA GAT GGT G -3′

### Polymerase chain reaction (PCR)

Genomic DNA was obtained from tissue sections from un-injected (control) and BPC- and eiBPCs-derived cardiac progenitor-injected mouse hearts. Sections were stained with anti-EGFP antibody to identify areas of injected cells present in the myocardium and these sections were used for PCR analysis. Additionally, random samples of recipient spleen, lung, liver and kidney were also used for DNA extraction to compare with responses occurring in the heart. PCR was conducted for EGFP to identify DNA of EGFP-labeled transplanted cells. DNA was extracted with QIAamp DNA micro kit (Qiagen); 100 ng DNA was mixed with primers for EGFP (Table 1). Final PCR products were run on agarose gel for detection of bands corresponding to EGFP.

### Differentiation of eiBPCs into cardiomyocytes

To determine differentiation potential of re-programmed BPCs, termed eiBPCs, into CMCs, the eiBPCs were cultured for 5 days in complete DMEM containing CMC induction medium with 5 ng/mL LIF and 3 ng/mL bone morphogenetic protein-2 (BMP-2) in 6-well culture plates (10^6^ cells per well) as well as 4-well chamber slides (10^4^ -cells per well) coated with 0.1% gelatin as in [13], [14]. Total cellular RNA was obtained from 6-well culture plates and qRT-PCR analysis of mRNA expression of CMC markers was carried out (Table 1); the markers used were GATA4, Nkx2.5, cardiotroponin T (CTT), CTI, α-sarcomeric actinin (α-SA), Mef2c, and MHC-α. For protein expression, cells were cultured for 10 days and protein expression was determined by immunochemical staining. For transplantation into myocardial infarction model, eiBPCs were cultured in CMC-conditioned medium for 24 hours to commit the cells towards CMC lineage [13] before transplantation.

### Immunofluorescence staining

Protein expression was evaluated by immunofluorescence staining as in [13], [14]. Briefly, cultured cells or cardiac tissue was rinsed once with phosphate-buffered saline (PBS) and fixed with 4% paraformaldehyde (Sigma) in PBS for 30 min, then rinsed three times with PBS, permeabilized with 0.3% Triton X-100 (Sigma) in PBS for 5 min, washed twice with PBS, and incubated overnight at 4°C with primary antibodies (against Oct4, Nkx2.5, Gata 4, MAP-2, MBP, GFAP, CTT, α-SA, GFP and Ki67) diluted with 1% FBS in PBS. After 3 washes with PBS, cells were incubated with specific secondary antibodies for 1 hour at 37°C, and cells were rinsed three times with PBS, stained with DAPI to visualize cell nuclei, rinsed 3 times with PBS, dried, and mounted in Vectashield mounting medium for fluorescent imaging. All immunofluorescence staining was photographed using either confocal and immunofluorescence microscope.

### Western blot analysis of AceH3K9, HDAC1 and LSD1 proteins

Immunoprecipitation and Western blot analyses of AceH3K9, HDAC1 and LSD1 proteins were performed in BPCs and eiBPCs as in [13] using antibodies purchased from Cell Signaling Technology, USA.

### Chromatin immunoprecipitation assay (ChIp)

ChIp assay was performed for the Oct4 promoter in BPCs, eiBPCs and mouse ESCs as described [14], [15] to identify transcription factors binding to Oct4 following treatment with Aza and TSA.

### Cell viability and proliferation assays

Viability and proliferation of the modified BPCs was evaluated by Trypan-blue exclusion and MTT assays, respectively. BPCs were cultured with different concentrations of Aza and TSA in 12-well culture plates for 24 or 48 hours, cells were harvested, exposed to Trypan blue solution (final concentration 0.1%), and number of viable (unstained) and non-viable (stained) cells were counted with a hemocytometer. Effects of Aza and TSA on BPC proliferation was determined by 3, 4, 5-(dimethylthiazol-2-yl) 2, 5- diphenyl tetrazolium bromide (MTT) assay according to manufacturer's instruction (Cell Proliferation Assay kit Promega). Briefly, 10^5^ cells were cultured in 96-well culture plate (200 ul/well) in DMEM containing 10% FBS in the presence of 0, 25, 50 and 100 ng/ml 5Aza or 0, 10, 25, 50 nm/ml TSA or combination of both for 48 hours at 37°C. MTT assay results were confirmed by manual cell counting under microscope.

### Acute myocardial infarction model in mice

All procedures were performed in accordance with guidelines of Institutional Animal Care and Use Committee of University of Illinois at Chicago (ACC No: 09-061 approved dated 05/08/2009). The study involved 8-week-old male C57BL/6J mice (n = 30); Jackson Laboratories). Mice underwent surgery to induce AMI by ligation of left anterior descending coronary artery [16], [17]. Animals subdivided into 3 groups received intramyocardial injection of 5×10^5^ GFP+cardiac progenitors derived from eiBPCs (treated for 24 hours with cardiomyocyte specific medium), control BPCs, or saline, respectively, in a total volume of 10 uL at 5 sites (basal anterior, mid-anterior, mid-lateral, apical anterior and apical lateral) in the peri-infarct area immediately after surgery.

### Left ventricular function and histology

Mice underwent echocardiography just before MI (base level) and at 1, 2, and 4 weeks after AMI as described [16], [17]. Trans-thoracic echocardiography was performed with a 6- to 15-MHz transducer (SONOS 5500, Hewlett Packard). Two-dimensional images were obtained in the parasternal long and short axis and apical 4-chamber views. M-mode images of the left ventricular short axis were taken just below the level of mid-papillary muscles. Left ventricular end-diastolic and end-systolic dimensions were measured and functional shortening was determined according to modified recommendations of American Society of Echocardiography. A mean value of 3 measurements was determined for each time point. On day 28 post-AMI, mice were euthanized and aortas were perfused with saline, hearts were removed and sliced into 4 transverse sections from apex to base and fixed with 4% paraformaldehyde, methanol, or frozen in OCT compound and sectioned into 5-µm thickness slices. Immunofluorescence staining was performed to determine myocyte and endothelial cell differentiation of transplanted cells. For assessment of fibrosis, tissues sections were frozen in OCT compound and sectioned for elastic tissue/trichrome to measure average ratio of the external circumference of fibrosis area to left ventricular area.

### Apoptosis assay

To assess survival of transplanted cells as well as effects of cell transplantation on survival of resident myocytes, apoptosis assay was performed on heart tissue collected at different time points. Cell death was assessed using the TUNEL kit according to manufacturer's instruction (Roche Applied Science). After TUNEL assay staining, sections were examined by confocal fluorescence microscopy.

### Measurement of chemokines and cytokines by transplanted cells

We measured mRNA levels of TNF-α, IL-1β, IL-6, MCP-1 and IL-10 in heart tissue bordering the infarcted myocardium as described [18].

### Staining for endothelial marker CD31

De-paraffinized heart tissue section slides were incubated for 30 min in peroxidase suppressor to quench endogenous peroxidase activity. After washing slides two times for 3 min with a wash buffer, blocking buffer was added and then incubated for 30 min. The tissue was incubated for 30 min with anti-CD31 antibody and slides were washed two times for 3 min with wash buffer. Again the tissues were incubated for 30 min with HRP-conjugated secondary antibody and slides were washed 3 times for 3 min each with wash buffer. Finally, the metal enhanced DAB Substrate was added and incubated until the desired staining was achieved.

### Statistical analysis

All experiments were made at least 3 times. Results are presented as mean+SEM. Comparisons were performed by ANOVA (GB-STAT; Dynamic Microsystems) or χ^2^ test for percentages. All tests were 2-sided, and probability value of <0.05 was considered as statistically significant.

## Results

### Expression of pluripotent genes in BPCs after treatment with Aza and TSA

**Figure 1:**
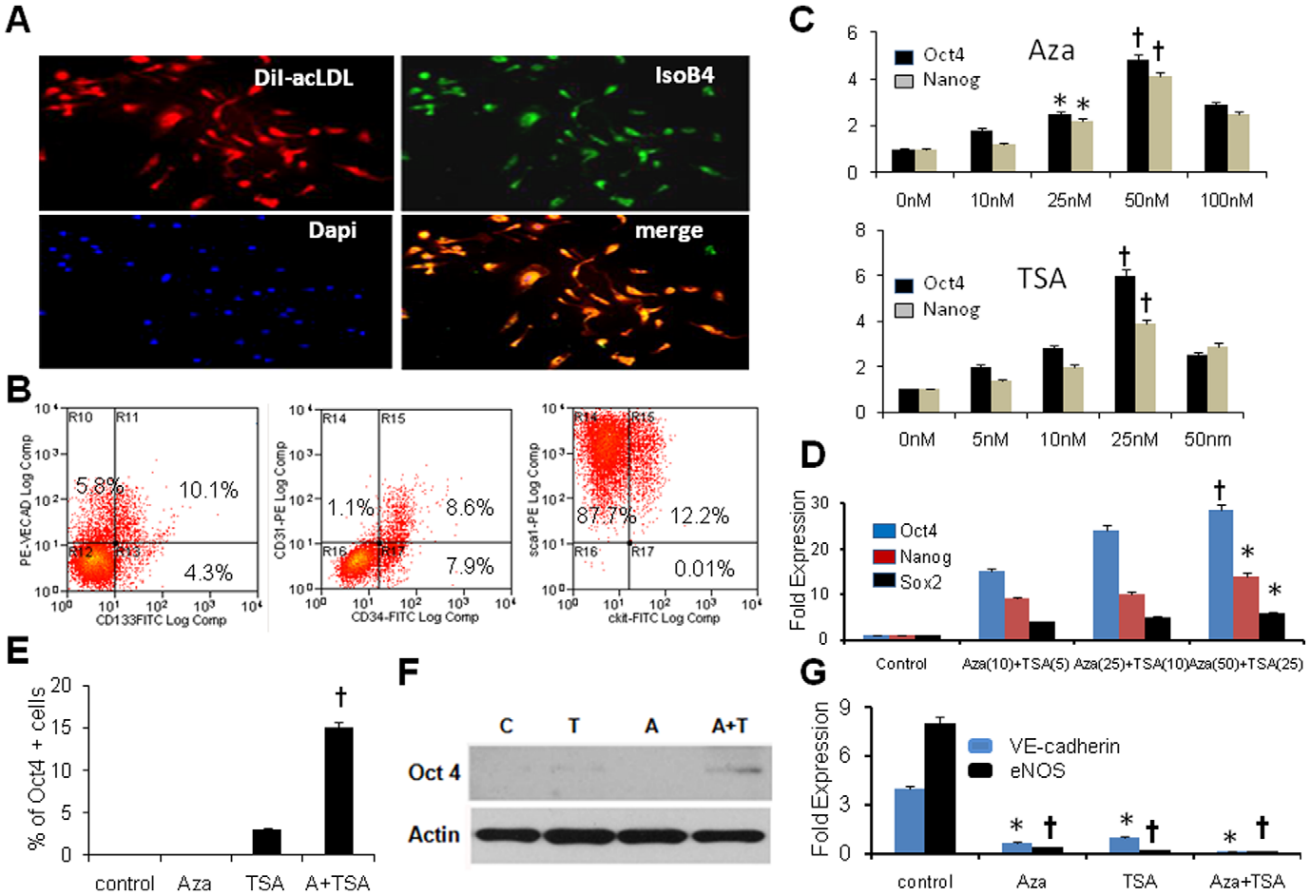
Characterization of Day-7 BPCs and their conversion into multipotent progenitor cells using Aza and TSA. Immunofluoresent analysis of BPCs stained with DiI-acLDL (red), Isolectin B4 (green), nuclei (blue,) and co-localized cells (yellow) (**A**); FACS analysis of BPCs for specific progenitor markers, (**B**); Dose-response relationship of Aza- or TSA-treated BPCs. RT-PCR analysis of Oct4 and Nanog transcripts after treatment of BPCs with Aza (0, 10, 25, 50, 100 nM) or TSA (0, 5, 10, 25, 50 nM) for 48 hours, (**C**); RT-PCR analysis of Oct4, Nanog and Sox2 transcripts after treatment of BPCs with combination of Aza (0, 10, 25, 50 nM) and TSA (0, 5, 10, 25 nM) for 48 hours, (**D**); Day-10 Oct4 protein expression by immunofluoresence, (**E**); Day-10 Oct4 protein expression by Western blotting, (**F**); RT-PCR analysis of endothelial markers eNOS and VE-cadherin transcripts after treatment of BPCs with Aza (50 nM) or TSA (25 nM) or combination of both drugs for 48 hours, (**G**); RT-PCR analysis; each bar represents mean ± S.E of 3 replicate experiments. Fold expression was calculated as ratio of experimental cell expression-to-expression in control cells. *p<0.01 vs. control, ^†^p<0.001 vs. control.

On day 7, BPCs obtained from C57BL/6J mouse bone marrow as described [12] were analyzed by immunofluorescence and flowcytometry (Figure 1A, B). Most cells were positive for Dil-acLDL and showed binding to fluorescein isothicyanate-conjugated *Banderia simplicifolia* lectin 1 (FITC-BS1). FACS analysis also showed BPCs were a heterogeneous population. They were positive for progenitor cell markers such as CD34 (17%), CD133 (10%), Sca1 (100%), and c-kit (12%) and some were also positive for endothelial cell markers CD31 (10%) and VE-cadherin (16%). BPCs were treated for 4 days with Aza alone, TSA alone, or both Aza and TSA at various concentrations. BPCs treated with Aza or TSA alone (Figure 1C) or with both agents in combination induced expression of Oct4, Nanog and Sox2 (Figure 1D, p<0.001 control vs. Aza+TSA treatment group). Maximum expression of these factors occurred at combined concentrations of 50 nM Aza and 25 nM TSA. Immunofluorescence analysis of Oct4 expression showed that BPCs treated with Aza+TSA showed significantly up-regulated expression of pluripotent genes at passage 0 (Figure 1E) but expression decreased markedly by passage 6 (data not shown). The expression of Oct4 gene transcript was supported by Oct4 Western blot protein expression data (Figure 1F). The results shown in Figure 1A-E demonstrate that pluripotent genes in BPCs were activated by co-treatment with Aza and TSA in a concentration-dependent manner. We also observed by qRT-PCR that BPCs treated with 50 nM Aza and 25 nM TSA resulted in silencing of eNOS and VE-cadherin genes compared to control cells (Figure 1G), suggesting that these drugs promoted de-differentiation of endothelial cells present in the BPC population.

### Aza and TSA do not affect BPC viability

**Figure 2:**
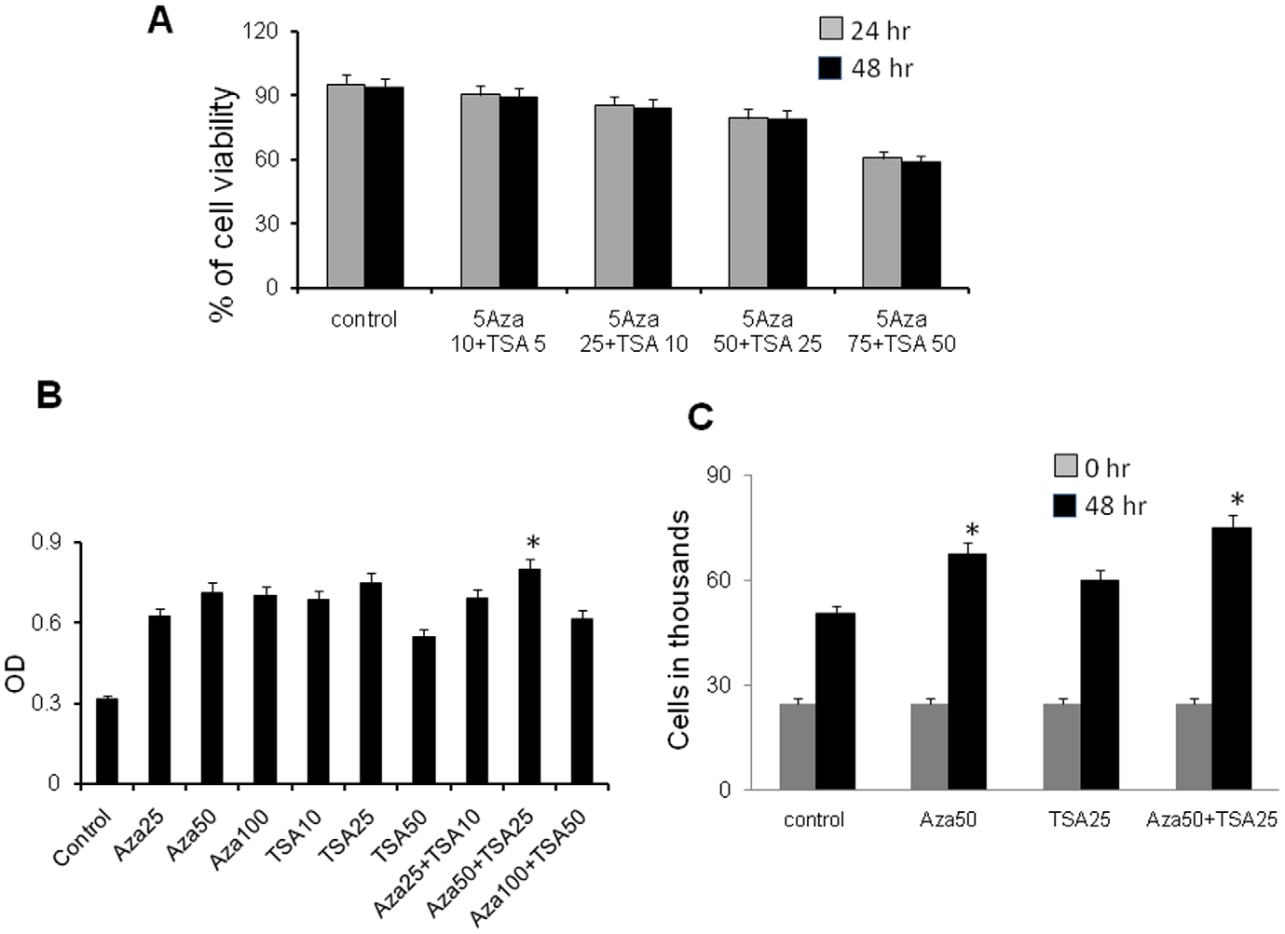
Viability and proliferative potential of Aza- and TSA-treated BPCs. Trypan blue dye exclusion viability assay in BPCs treated with 5Aza and TSA **(A)**. Proliferation of treated BPCs assessed by MTT method **(B)**. Cell proliferation assessed by hemocytometer cell counting after 48 hours **(C)**. Each bar represents mean ± S.E of 3 replicated experiments. *p<0.05 vs. control.

BPCs were cultured with different concentrations of Aza and TSA in 12-well culture plates for 24 or 48 hours, cells were harvested, exposed to Trypan blue solution (final concentration 0.1%), and number of viable (unstained) and non-viable (stained) cells were counted with a hemocytometer. Treatment with up to 50 nM Aza and 25 nM TSA had no effect on cell viability (Figure 2A, p = 0.09). The modified BPCs also showed normal ability to proliferate (Figure 2B, C p<0.05)

### Epigenetic changes in Aza- and TSA-treated BPCs

**Figure 3:**
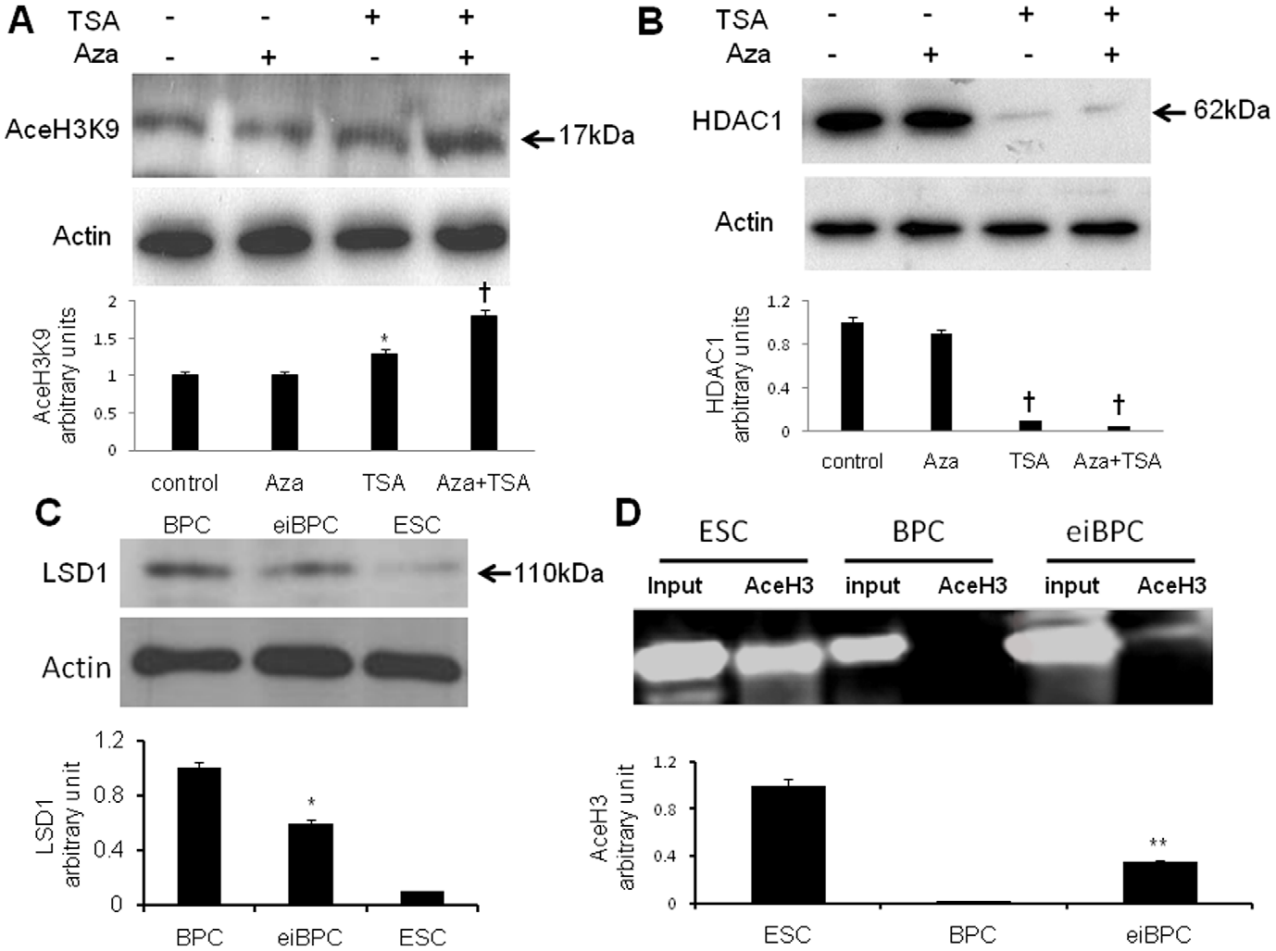
Modification of Aza- and TSA-treated BPCs reflected in expression of AceH3K9 and LSD1 and acetylation of Oct4. Treatment of BPCs with Aza + TSA (eiBPCs) shows increased AceH3K9 expression (**A**), decreased HDAC1 expression (**B**), and decreased LSD1 expression (**C**) assessed Western blotting. Promoter of Oct4 was analysed by ChIp using anti-AceH3K9 antibody or anti-IgG control antibody. Acetylation status of histone H3 showed that Oct4 promoter was highly acetylated in mouse ESCs (positive control), was not acetylated in untreated BPCs, but was significantly acetylated in Aza- and TSA-treated BPCs (eiBPCs) (**D**). Results show representative data of n = 3. *P<0.05 vs. control, **p<0.01 vs. control, ^†^p<0.005 vs. control.

We determined whether Aza and TSA treatment of BPCs altered acetylation and methylation status of AceH3K9, HDAC1, and LSD1 proteins involved in induction of multipotency [19]. BPCs were treated with 5Aza and TSA, either alone or in combination for 2 days. As shown in Figure 3A, expression of acetylated H3 lysine9 (AceH3K9) protein was significantly increased in BPCs treated with TSA alone (p<0.01) or with Aza plus TSA (p<0.005) compared to controls. HDAC1 protein expression decreased after treatment of BPCs with either TSA alone (p<0.005) or combination of Aza+TSA (p<0.005) compared to control cells (Figure 3B). Compared to control BPCs, the treated BPCs displayed decreased LSD1 expression (p<0.05) (Figure 3C).

We also determined whether Aza and TSA induced changes in AceH3K9 protein interaction with Oct4 promoter [14] to assess whether these agents can epigenetically modify BPCs. As shown in Figure 3D, Oct4 promoter was highly acetylated in positive control mouse ESCs, whereas acetylation of H3 region in Oct4 promoter was not detected in untreated BPCs but it was much evident in the treated BPC (p<0.05).

### Differentiation of Aza- and TSA-modified BPCs into cardiomyocytes

**Figure 4:**
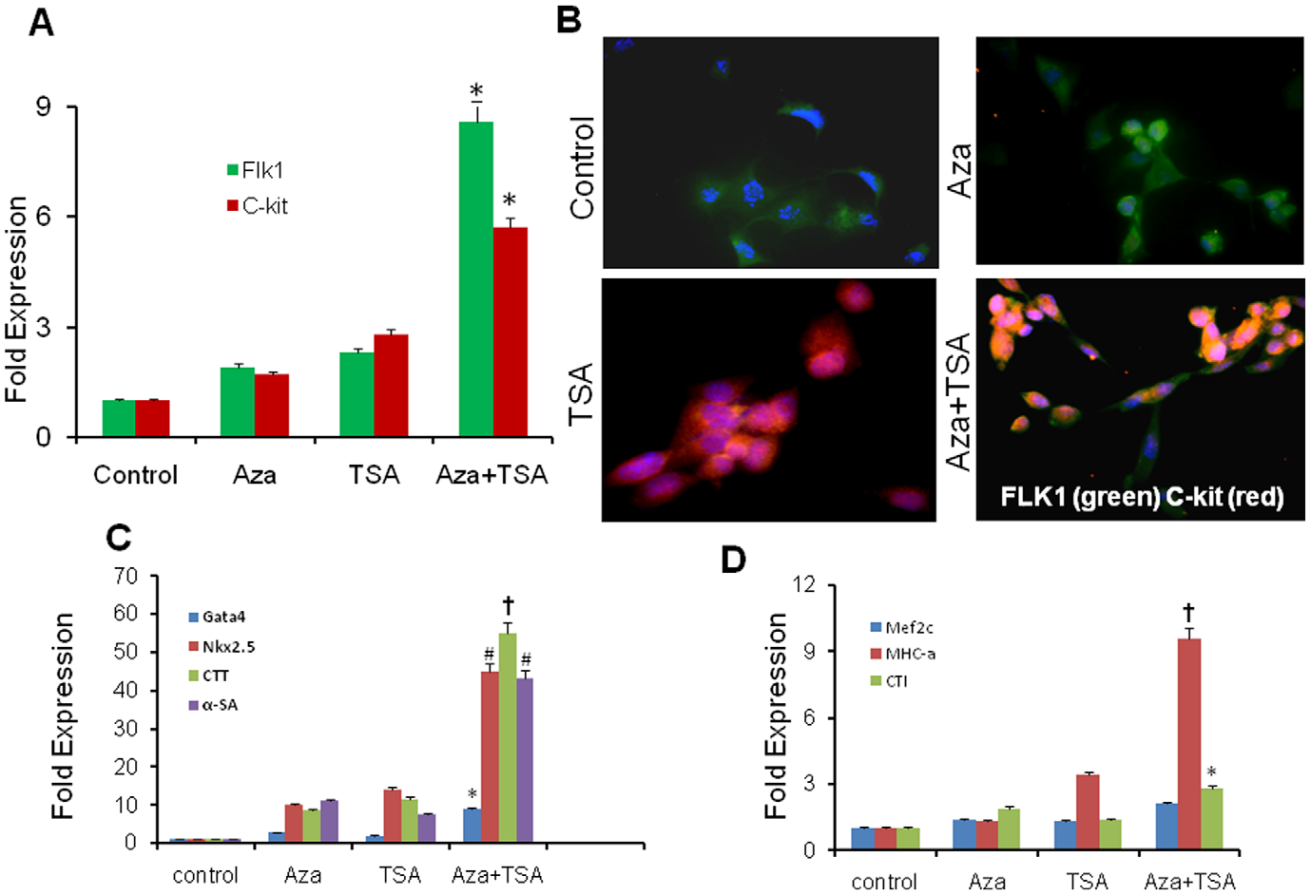
Conversion of eiBPCs into myocytes. eiBPCs were cultured in conditions favoring their transition into cardiac progenitors (see Method), and RT-PCR analysis was carried out. Results show Flk1 and c-kit transcripts after 24 hours in cardiomyocyte differentiation medium (**A**). Protein expression of Flk1 (green) and c-kit (red) after 24 hours in cardiomyocyte differentiation medium analyzed by immunofluorescence showing higher number of Flk1+/c-kit+ cells (yellow). Greater number of cardiac progenitor cells was evident in BPCs treated with combination of Aza and TSA vs. treatment with each agent alone or in control cells (**B**). Differentiated cardiac progenitor cells derived eiBPCs at 5 days after culture in myocyte differentiation medium expressed the cardiomyocyte specific gene transcripts Gata4, Nkx2.5, CTT and α-SA (**C**) and Mef2c, MHC-α and CTI as determined by qRT-PCR (**D**). Response was evident only in BPCs treated with Aza (50 nM) + TSA (25 nM) for 5 days. Each bar represents mean ± S.E of 3 replicated experiments. Image magnification 400X. *p<0.01 vs. control, #p<0.005 vs. control, ^†^p<0.001 vs. control.

To address whether Aza and TSA modified BPCs (eiBPCs) could give rise into myocyte progenitor lineage cells and cardiomyocytes, eiBPC were cultured for 5 or 10 days in CMC induction medium. We observed that cardiac progenitor cell markers Flk1 and c-kit were up-regulated as early as 24 hr after combined treatment with Aza and TSA compared to control or BPC treated with Aza or TSA alone (Figure 4A, B). Cardiomyocyte specific markers, GATA4, Nkx2.5, CTT, α-sarcomeric actinin, Mef2c, MHC-α and CTI were up-regulated after combined treatment of Aza and TSA at day 5 compared to BPCs treated with Aza or TSA alone (Figure 4C, D; P<0.001 control vs. cells treated with both Aza and TSA). eiBPCs or cardiac progenitor cells derived from eiBPCs failed to differentiate into endodermal lineage of cells and did not form teratomas in SCID mice (data not shown) suggesting that eiBPCs or cardiac progenitors derived from eiBPCs had been partially re-programmed but were not pluripotent.

### Intracardiac injection of eiBPC-derived cardiac progenitors improves post-infarction left ventricular function

**Figure 5:**
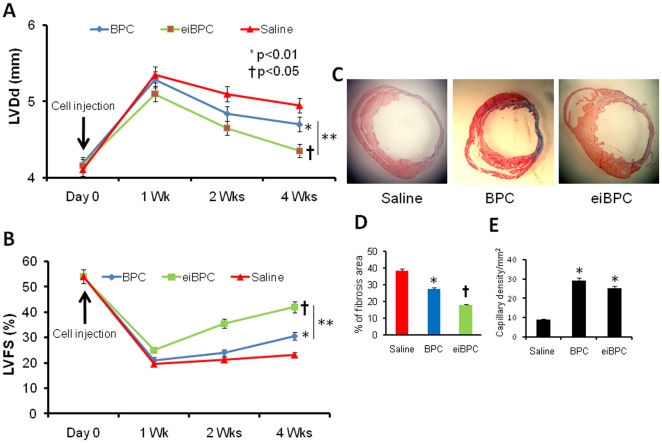
Intracardiac injection of eiBPC-derived cardiac progenitors improves left ventricular (LV) function and decreases myocardial fibrosis in mouse AMI model. Echocardiographic LV function parameters measured 1, 2 and 4 weeks after AMI and cell transplantation showed greater recovery in LV diastolic dimension (LVDd) and LV fractional shortening (LVFS) after transplantation of cardiac progenitors derived from eiBPCs vs. BPC transplantation and saline injected controls, (**A, B**); Trichrome staining of cardiac tissue show markedly enlarged LV in saline and was less enlarged after BPC transplantation and transplantation of cardiac progenitors derived from eiBPCs (**C**); Quantification of relative fibrotic areas in hearts in each group (**D**). Quantification of capillary density measured by CD31 DAB staining showing greater staining after transplantation of BPCs and cardiac progenitors derived from eiBPCs relative to saline control group (**E**). Each bar represents mean ± S.E of 3 replicated experiments. *p<0.05 vs. saline, **p<0.05 vs. BPCs, ^†^p<0.001 vs. saline.

We used a standardized mouse AMI model [16], [17] to test the efficacy of cardiac progenitors derived from eiBPCs (as described Methods) in improving LV function. Physiological assessment of LV function was made before AMI and on days 7, 14 and 28 post-AMI. The day 28 post-AMI echocardiography showed significantly improved LV diastolic dimensions (LVDd) and percentage of LV fractional shortening (%LVFS) in mice transplanted with eiBPCs and BPCs compared to saline control (Figure 5A, B). Mouse hearts treated cardiac progenitors derived from eiBPCs had better functional outcome post-AMI than BPC treatment alone. Myocardial regeneration was only seen in the infarcted myocardium in hearts treated with Aza- plus TSA-modified BPCs. Masson's trichrome-stained sections showed markedly enlarged LV in saline-injected AMI mice and LVs were less enlarged in mouse hearts injected with Aza- plus TSA-modified BPCs (Figure 5C). Heart sections were also quantified for % LV fibrosis as the ratio of length of fibrosis scar-to-LV circumference. We observed marked reduction in LV fibrosis in hearts treated with eiBPC-derived cardiac progenitors compared with hearts treated with BPCs or saline (Figure 5D). We also examined the capillary density in ischemic area by DAB staining using anti-CD31 antibody marking for capillaries (Figure 5E). Five randomly chosen sections were quantified in microscopic visual fields on each slide and mean was calculated. We observed a closely similar number of capillaries in BPC-transplanted hearts (28±3) and eiBPC-derived cardiac progenitor-transplanted hearts (25±2) (Figure 5E, P = 0.06), suggesting that propensity of BPCs to differentiate into endothelial cells was similar to cardiac progenitors from eiBPCs.

### Myocardiac injection of eiBPC-derived cardiac progenitor cells suppresses inflammation

**Figure 6:**
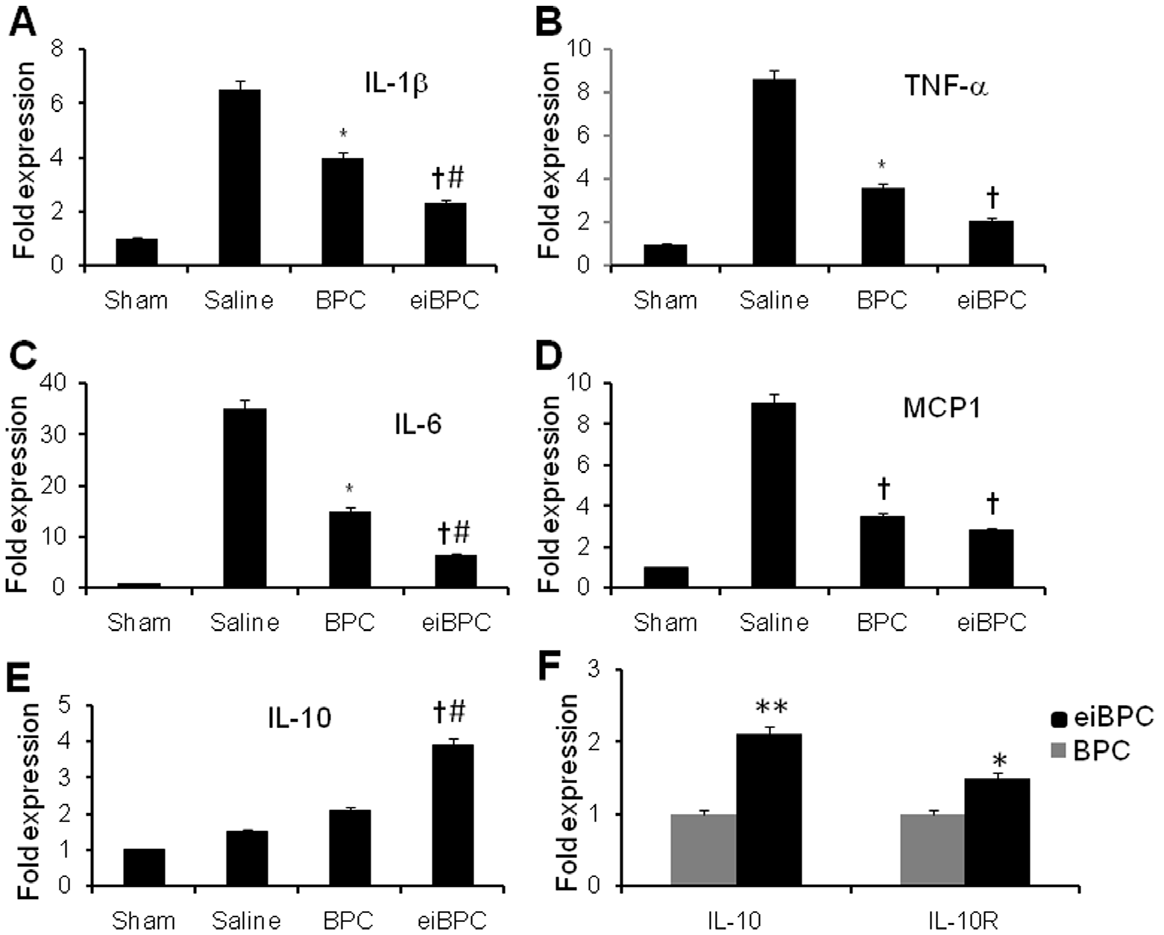
Transplantation of eiBPC-derived cardiac progenitors suppresses inflammation. mRNA expression of (**A**) IL-β; (**B**) TNF-α; (**C**) IL-6; (**D**) MCP1; (**E**) IL-10 at the infarct border zone. *p<0.01 vs. saline, #p<0.05 vs. BPCs, ^†^p<0.005 vs. saline. Cardiac progenitors derived from eiBPCs significantly expressed IL-10 and IL-10R transcripts. Cardiac progenitors were obtained by culturing eiBPCs for 24 hours in cardiomyocyte conditioned medium (as described in Methods) and cells were obtained for qRT-PCR analysis. IL-10 and IL-10R mRNA were in cardiac progenitor cells derived from eiBPCs relative to BPCs *p<0.05 vs. BPCs, * *p<0.03 vs. BPCs (**F**). Each bar represents mean ± S.E of 3 replicate experiments.

To determine effects of cardiac progenitors derived from eiBPCs on expression of pro-inflammatory and anti-inflammatory cytokines and chemokines (TNF-α, IL-1β, IL-6, MCP-1 and IL-10) in the myocardium at 3 days post-AMI was assessed by qRT-PCR. Expression of pro-inflammatory cytokines and chemokines was increased in myocardium of saline-treated and was decreased in BPC-treated (Figure 6A-D, p<0.01 vs. saline) and eiBPC-derived cardiac progenitor-treated myocardium (Figure 6A-D, p<0.005 vs. saline, P<0.05 vs. BPC). The anti-inflammatory cytokine IL-10 mRNA expression was increased in eiBPC-derived cardiac progenitor group compared to saline- or BPC-treated group (Figure 6E, p<0.01 vs. saline, p<0.05 vs. BPC) treated myocardium. Even in non-injected eiBPC-derived cardiac progenitors, there was increased IL-10 and IL-10R mRNA expression vs. BPCs (Figure 6F).

### Myocardial injection of cardiac progenitors derived from eiBPCs induces expression of cardiac cells and new vessel formation

**Figure 7:**
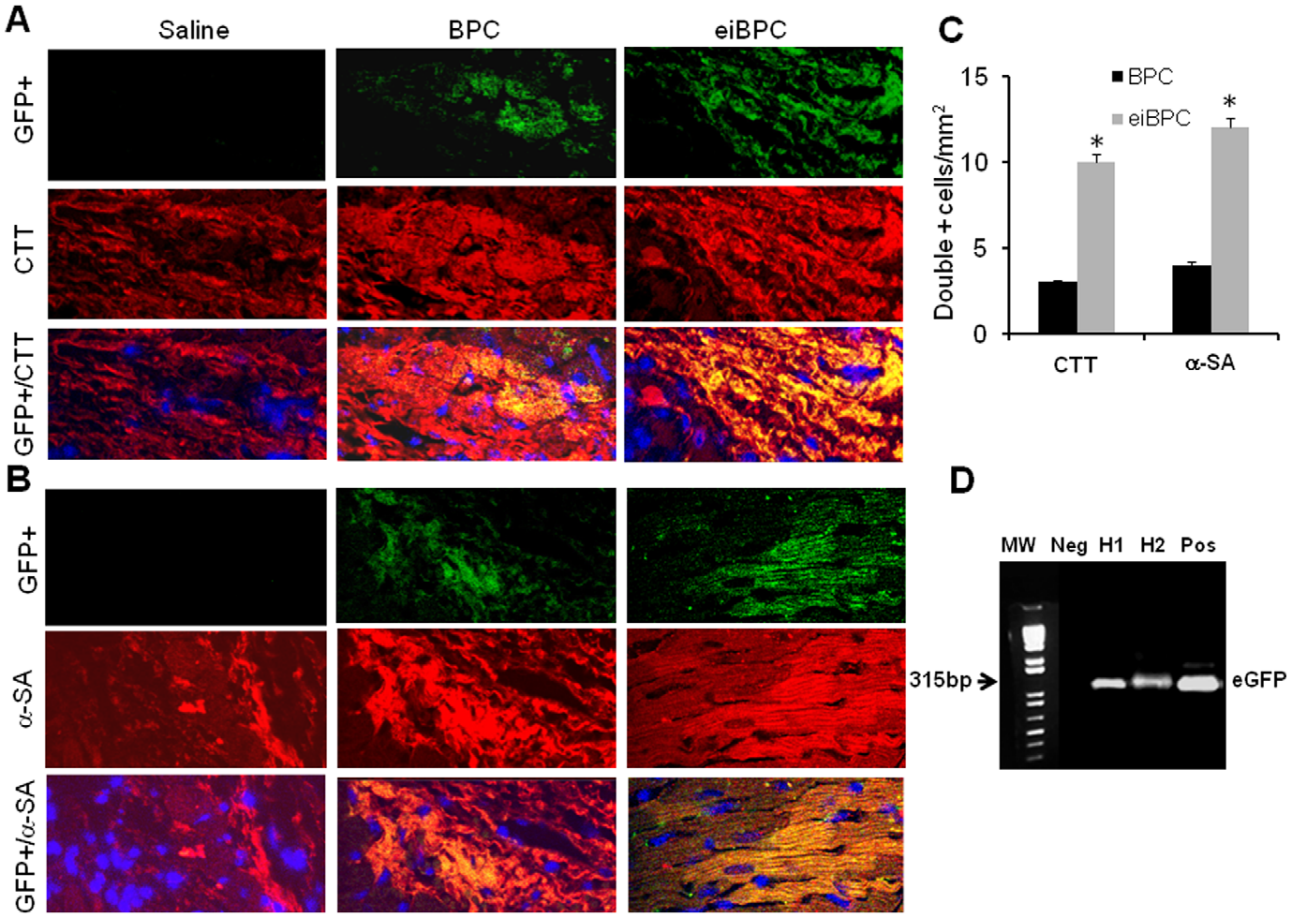
Differentiation of transplanted cardiac progenitor cells derived from eiBPCs into cardiomyocyes in mouse hearts. Co-localization of transplanted GFP+ (green) and cardiomyocyte-specific marker cardio-troponin T (CTT-red) (**A**) and α-SA-red (**B**); DAPI-blue. Differentiation was into CTT+ and α-SA+ cells was only seen in mouse hearts transplanted with eiBPC-derived cardiac progenitor cells as compared to hearts transplanted with BPCs or injected with saline. Quantification of GFP+/CTT+ and GFP+/α-SA+ cells in the heart (**C**). eGFP DNA sequences determined by PCR in heart tissue. Neg, DNA from wild-type heart; Pos, DNA from eGFP transgenic mice; H1, DNA from BPC-transplanted heart; H2, DNA from eiBPC cardiac progenitor-transplanted heart (**D**). Each bar represents mean ± S.E. Image magnification 630X. *p<0.01 vs. BPC. Results of representative of n = 3.

Immunofluorescence staining was performed to determine *in situ* differentiation of transplanted GFP+eiBPC-derived cardiac progenitors in the myocardium. The 28 days post-cell injected heart tissue sections were co-stained with CMC tissue markers cardiotroponin T (CTT) (Figure 7A) and α-SA (Figure 7B). The GPF+ cells (green) expressing the two CMC proteins (red) were double positive (yellow) merged images indicating the co-localization and differentiation of injected cells. Double positive cells were counted at 5 randomly chosen fields on each slide, and the mean is presented as double positive cells/mm2 (Figure 7C). As shown in Figure 7, transplantation of cardiac progenitors derived from eiBPCs induced differentiation of progenitor cells to CMCs *in situ*; transplantation of eiBPC-derived cardiac progenitors also showed greater number of cells co-expressing the CMC markers than transplantation of BPCs alone (p<0.01). Genomic DNA obtained from un-injected hearts and hearts from BPC-injected hearts and eiBPC-derived cardiac progenitor-injected hearts was also used for PCR analysis. Additionally, samples of spleen, lung, liver and kidney were used for DNA extraction. PCR for EGFP used to identify DNA of GFP-labeled transplanted cells in tissue. The bands on agarose gel corresponding to eGFP showed that green cells were present in heart tissue (Figure 7D) and bands from the other organs did not express GFP DNA (data not shown).

**Figure 8:**
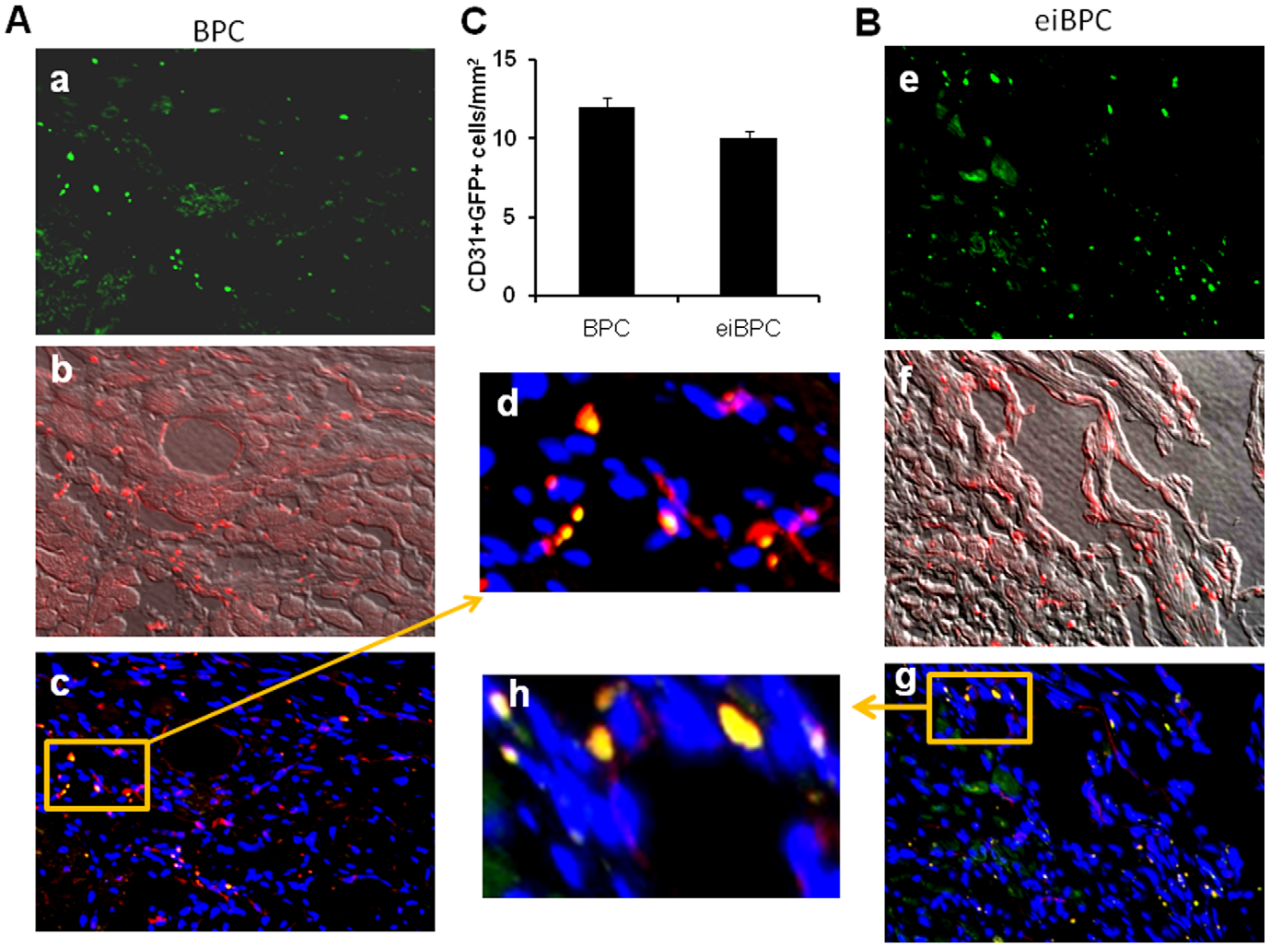
Neovascularization after transplantation of cardiac progenitor cells from eiBPCs into mouse hearts. (**A**). GFP+CD31+ double positive cells were observed in myocardial sections obtained from mice transplanted with BPCs (a-GFP+ transplanted cells, b-Phase contrast image merged with CD31+ cells, c- double positive cells merge with DAPI, d- higher magnification) and (**B**). cardiac progenitors derived from eiBPC (e-GFP+ transplanted cells, f- Phase contrast image merged with CD31+ cells, g- double positive cells merge with DAPI-higher magnification). **C**), endothelial differentiation in myocardial tissues was quantified by counting double positive CD31+GFP+ cells in microscopic visual field (expressed as mm^2^). Number of GFP+CD31+ double positive cells was observed in following BPC transplantation (12±2) and transplantation of cardiac progenitors derived from eiBPCs (10±1, *P* = 0.09). Image magnification 200X.

As shown in Figure 8, differentiation into GFP+CD31+ double positive cells was marginally increased in myocardial sections obtained from mouse hearts transplanted with BPCs (Figure 8A) compared to larger increase in mouse hearts transplanted with eiBPC-derived cardiac progenitors (Figure 8B). Endothelial differentiation in myocardial tissue was quantified by counting CD31+GFP+ cells (Figure 8C). Number of GFP+CD31+ cells similarly increased in both BPC-transplanted hearts (12±2) and eiBPC-derived cardiac progenitor transplanted hearts (10±1, *P* = 0.09).

**Figure 9:**
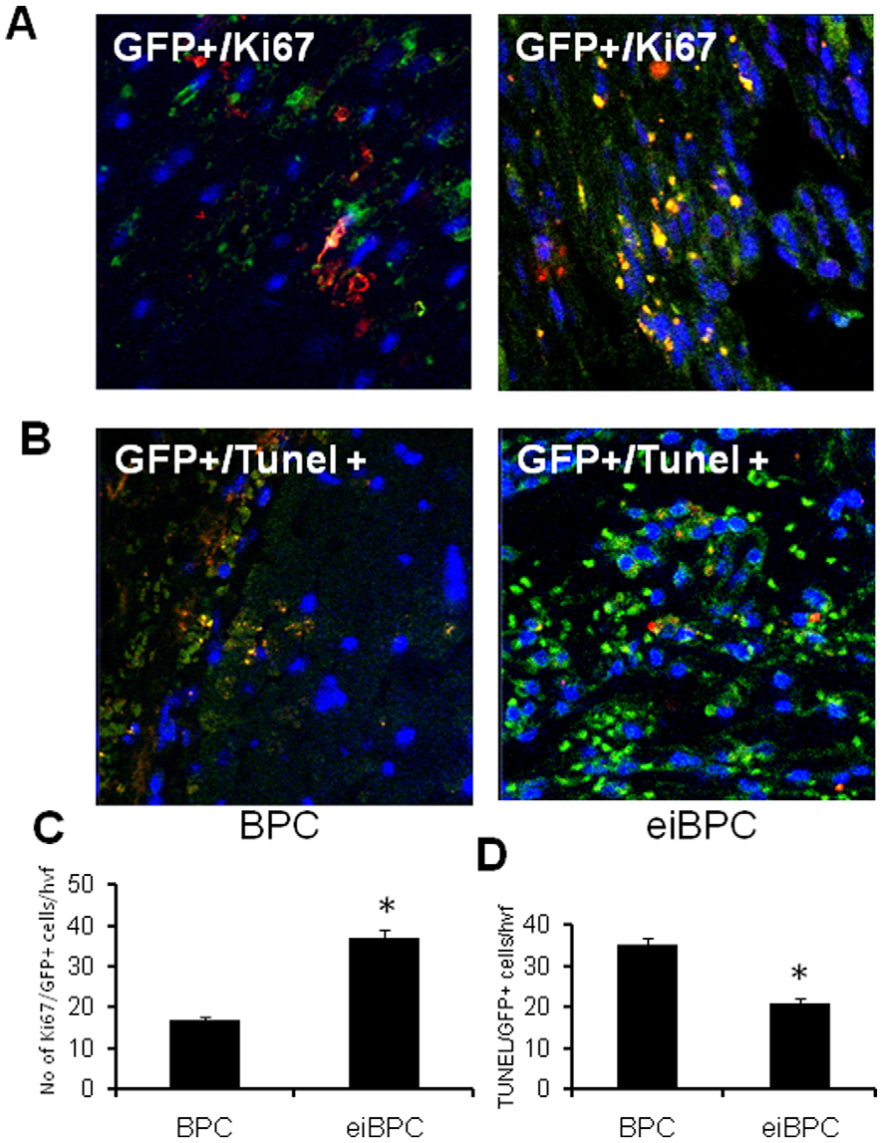
Transplantation of cardiac progenitors derived from eiBPCs increases proliferation and decreases cell death in ischemic myocardium. We determined the number of proliferating GFP+/Ki67+ (yellow) (Ki67 - red, DAPI- blue) cells following transplantation of BPCs and cardiac progenitors derived from eiBPCs (**A**). Number of double positive cells (Ki67 and GFP- yellow) was counted using an immunofluoresence microscope (**C**). Cell death was assessed by TUNEL staining (**B**). Number of TUNEL+ cells was assessed by double positive of GFP+/TUNEL+ (yellow), TUNEL-red, DAPI-blue was also counted (**D**). Each bar represents mean ± S.E of 3 replicated experiments. *p<0.05 vs. BPCs.

Transplanted eiBPC-derived cardiac progenitors also showed evidence of proliferation *in vivo*, as determined by co-localization of GFP+ cells with nuclear proliferation antigen, Ki67, seen at day 28 post-transplantation (Figure 9A, C), whereas fewer GFP+Ki67+ cells were seen in BPC-transplanted hearts. This proliferative effect is consistent with the *in vitro* data above (Figure 2). In addition the number of apoptotic cells was less in eiBPC-derived cardiac progenitor transplanted group than in BPC-transplanted group (Figure 9 B, D).

## Discussion

Progress has been made recently in inducing cardiac regeneration aimed at repairing the damaged cardiac tissue [5], [20]. BPCs and EPCs have been extensively used in these studies [21]; however, the results have been variable and often improvements have been short-lived perhaps due to waning effects of the released humoral mediators from the transplanted cells and tissue and failure of effective engraftment [5]. In addition, it is not always clear whether transplanted cells differentiate into cardiac or endothelial cells and thus whether they have the capability to regenerate injured tissue. Cells such as BPCs also consist of heterogeneous population of bone marrow cells containing mesenchymal cells, EPCs, and mature endothelial cells [17]. These cells may not have trans-differentiation potential of cardiac progenitor cells [22], [23]. Here we demonstrate for the first time to our knowledge of the value of epigenetic modification of BPCs, which induces the BPCs to de-differentiate into multipotential cells, which we termed eiBPCs. Transplantation of cardiac progenitor cells derived from eiBPCs into mouse hearts induced significantly greater cardiac protection and myocyte regeneration in mice after myocardial infarction over that seen with BPCs alone. Based on these results, we posit that cardiac progenitor cells derived from epigenetic modification of autologous BPCs have promise for treating ischemic heart disease. These findings thus raise the possibility of converting BPCs into multipotent eiBPCs as a source of cardiac regenerating cells.

Epigenetic changes are defined as modifications of DNA or chromatin that do not involve alterations of the DNA sequence or genetic deletions [7], [24]. Recent studies into the mechanisms by which nuclear cloning and somatic-ESC fusion induce epigenetic changes in somatic cell nuclei suggest that the somatic cell chromatin is remodeled through chromatin condensation, DNA methylation/de-methylation, and histone modifications (acetylation/de-acetylation, phosphorylation, and methylation/de-methylation) [25], [26]. These histone and DNA modifications are functionally linked [7], [8]. DNA de-methylation/methylation is an essential epigenetic process required for gene activation or inactivation during development [27]. A change in DNA methylation pattern is also a principal epigenetic event underlying de-differentiation of somatic cells [28]–[30]. DNA methylation most often occurs on 5′-CpG-3′ dinucleotides [31]. DNA methyl transferase enzymes (dnmt 1, dnmt3a, dnmt 3b) attach a methyl group to the 5^th^ carbon position of cytosine residues in the CG dinucleotide [30], the protein condenses into a protein complex consisting of methyl-binding proteins (MBD), HDACs, and repressor proteins at the methylated CpG sites [29], which leads to inhibition of gene expression [32]. In addition to DNA methylation, post-translational modifications of histone proteins regulate gene activity by modulating chromatin structure [33]. Acetylation of histone H3 and H4 generally correlates with gene activation whereas de-acetylation correlates with gene silencing [34].

Gene silencing is achieved by DNA methylation and histone deacetylation at the promoter region of genes [35] whereas gene activation is mediated by DNA demethylation and histone acetylation [36]. DNA demethylation and methylation are mediated and balanced by DNA demethyltransferases (DeMT) and DNA methyltransferases (Dnmts) respectively. In this study, we used Aza, an inhibitor for Dnmts, which blocks DNA methylation and thus promotes demethylation [6]. Likewise histone acetylation and deacetylation is mediated and balanced by the enzymes histone acetyltransferases (HATs) and histone deacetylases (HDACs), respectively. Here we also used TSA, a general inhibitor for HDACs that blocks deacetylation and promotes histone acetylation [6]. Our data show that BPCs treated with Aza plus TSA in a dose-dependent manner activated the pluripotent genes Oct4, Nanog and Sox2 and resulted in expression of these proteins [37]. However, we do not think that these cells became totipotent as defined earlier [37], since transplantation of these cells did not result in the formation of teratomas.

Transcriptional activation of pluripotent genes such as Oct4 requires acetylation of histone H3 at lysine 9 (aceH3K9), whereas histone deacetylation by HDACs 1, 2, 3, and 8 induces closed chromatin confirmation leading to repression of gene activity [38], [39]. Transcriptional activation is also modulated by histone demethylase activity of LSD1, a homolog of nuclear amine oxidase; however, depending on the cellular context, LSD1 can either repress transcription as a component of the CoREST transcriptional co-repressor complex [40], [41] or activate transcription by functioning as a co-activator [40]. Our results show that treatment of BPCs with Aza plus TSA induced modifications resulting in expression of genes associated with a multipotent phenotype. We observed decreases in HDAC1 and LSD1 protein expression and increase in AceH3K9 protein expression indicative of epigenetic changes at the level of histones. We also observed increased aceH3K9 protein expression and its interaction with Oct4 promoter demonstrating that Oct4 promoter region was acetylated in the Aza- and TSA-treated BPCs. Thus, the chromatin modifying agents Aza and TSA induced epigenetic modification of BPCs at least in part by histone modifications and chromatin remodeling leading to the activation of dormant pluripotent genes.

We further transplantation of cardiac progenitor cells derived from the modified BPCs induced cardiomyogenesis and angiogenesis in infarcted hearts. As new vessel formation following cardiac progenitor cell transplantation was similar to that seen with BPC transplantation, it appears that angiogenesis is due to EPCs present in the transplanted cell population that were not fully committed to their myocyte fate.

Importantly, we did not observe teratoma formation following injection of cardiac progenitors. This is consistent with the use of cardiac progenitors derived from epigenetically multipotent BPCs rather fully de-differentiated iPSCs [37], [42], [43]. We did not examine the *in situ* electrophysiological properties of the regenerated CMCs; thus, although LV function was improved after cell transplantation, we do know whether the myocytes formed a proper syncytium. In addition, we observed only up to 5% of transplanted cells were retained in the myocardium at day 28 in absence of significant apoptosis, suggesting that the injected cells may have transitioned into myocytes during this period. Also it is possible that secretion of factors by transplanted cells or host tissue may contribute to the improvement in cardiac function. We observed the production of IL-10, an anti-inflammatory cytokine, in cardiac tissue after transplantation, which could improve LV function and remodeling via activation of STAT3 and suppression of p38 MAPKs [18].

Our observations are consistent with recent studies showing the feasibility of reprogramming of somatic cells into the CMC lineage [44]. A recent study also demonstrated reprogramming of fibroblasts into multi-lineage hematopoietic progenitor cells and mature blood cells without establishing totipotency [45]. They accomplished this by ectopic expression of Oct4-activated hematopoietic transcription factors and treatment with specific cytokines. We showed differentiation of BPCs into cardiac progenitor cells expressing the CMC markers Gata4, Nkx2.5, CTT, and α-SA is possible using the chromatin modifying agents Aza and TSA.

In summary, we showed that treatment of BPCs with chromatin modifying agents Aza and TSA induced expression of pluripotent genes Sox2, Oct4 and Nanog in BPCs, which can then be further programmed to generate cardiac progenitor cells that give rise to cardiac and endothelial cells *in situ*. Transplantation of these epigenetically reprogrammed cardiac progenitors cells induced cardiomyogenesis and also improved LV function of infarcted mouse hearts suggesting the utility of this approach. We used available chromatin modifying drugs to achieve multipotency of BPCs suggests clinical applicability and value of this cell transplantation approach.

## References

[1] Losordo DW, Dimmeler S (2004). Therapeutic angiogenesis and vasculogenesis for ischemic disease: part II: cell-based therapies.. Circulation.

[2] Jujo K, Ii M, Losordo DW (2008). Endothelial progenitor cells in neovascularization of infarcted myocardium.. J Mol Cell Cardiol.

[3] Murry CE, Soonpaa MH, Reinecke H, Nakajima H, Nakajima HO (2004). Haematopoietic stem cells do not transdifferentiate into cardiac myocytes in myocardial infarcts.. Nature.

[4] Balsam LB, Wagers AJ, Christensen JL, Kofidis T, Weissman IL (2004). Haematopoietic stem cells adopt mature haematopoietic fates in ischemic myocardium.. Nature.

[5] Perin EC, Silva GV (2010). Cell-based therapy for chronic ischemic heart disease-a clinical perspective.. Cardiovasc Ther.

[6] Ishimaru N, Fukuchi M, Hirai A, Chiba Y, Tamura T (2010). Differential epigenetic regulation of BDNF and NT-3 genes by trichostatin A and 5-aza-2′-deoxycytidine in Neuro-2a cells.. Biochem Biophys Res Commun.

[7] Trojer P, Reinberg D (2006). Histone Lysine Demethylase and their impact on epigenetics.. Cell.

[8] Klose RJ, Bird AP (2006). Genomic DNA methylation: the mark and its mediators.. Trends Biochem Sci.

[9] Ruau D, Ensenat-Waser R, Dinger TC, Vallabhapurapu DS, Rolletschek A (2008). Pluripotency associated genes are reactivated by chromatin-modifying agents in neurosphere cells.. Stem Cells.

[10] Araki H, Yoshinaga K, Boccuni P, Zhao Y, Hoffman R (2007). Chromatin-modifying agents permit human hematopoietic stem cells to undergo multiple cell divisions while retaining their repopulating potential.. Blood.

[11] Makino S, Fukuda K, Miyoshi S, Konishi F, Kodama H (1999). Cardiomyocytes can be generated from marrow stromal cells in vitro.. J Clin Invest.

[12] Asahara T, Masuda H, Takahashi T, Kalka C, Pastore C (1999). Bone marrow origin of endothelial progenitor cells responsible for postnatal vasculogenesis in physiological and pathological neovascularization.. Circ Res.

[13] Rajasingh J, Bord E, Hamada H, Lambers E, Qin G (2007). STAT3-dependent mouse embryonic stem cell differentiation into cardiomyocytes: analysis of molecular signaling and therapeutic efficacy of cardiomyocyte precommitted mES transplantation in a mouse model of myocardial infarction.. Circ Res.

[14] Rajasingh J, Lambers E, Hamada H, Bord E, Thorne T (2008). Cell-free Embryonic Stem Cell Extract-mediated derivation of Multipotent Stem Cells from NH3T3 Fibroblasts for Functional and Anatomical Ischemic Tissue Repair.. Circ Res.

[15] Kishore R, Qin G, Luedemann C, Bord E, Hanley A (2005). The cytoskeletal protein ezrin regulates EC proliferation and angiogenesis via TNF-alpha-induced transcriptional repression of cyclin A.. J Clin Invest.

[16] Kusano KF, Pola R, Murayama T, Curry C, Kawamoto A (2005). Sonic hedgehog myocardial gene therapy: tissue repair through transient reconstitution of embryonic signaling.. Nat Med.

[17] Iwakura A, Shastry S, Luedemann C, Hamada H, Kawamoto A (2006). Estradiol enhances recovery after myocardial infarction by augmenting incorporation of bone marrow-derived endothelial progenitor cells into sites of ischemia-induced neovascularization via endothelial nitric oxide synthase-mediated activation of matrix metalloproteinase-9.. Circulation.

[18] Krishnamurthy P, Rajasingh J, Lambers E, Qin G, Losordo DW (2009). IL-10 Inhibits Inflammation and Attenuates Left Ventricular Remodeling After Myocardial Infarction via Activation of STAT3 and Suppression of HuR.. Circ Res.

[19] Nakayama J, Rice JC, Strahl BD, Allis CD, Grewal SIS (2001). Role of Histone H3 Lysine 9 methylation in epigenetic control of heterochromatin assembly.. Science.

[20] Gupta R, Losordo DW (2010). Challenges in; the translation of cardiovascular cell therapy.. J Nucl Med.

[21] Beitnes JO, Hopp E, Lunde K, Solheim S, Arnesen H (2009). Long-term results after intracoronary injection of autologous mononuclear bone marrow cells in acute myocardial infarction: the ASTAMI randomized, controlled study.. Heart.

[22] Schmidt-Lucke C, Rossig L, Fichtlscherer S, Vasa M, Britten M (2005). Reduced number of circulating endothelial progenitor cells predicts future cardiovascular events: proof of concept for the clinical importance of endogenous vascular repair.. Circulation.

[23] Urbich C, Dimmeler S (2005). Risk factors for coronary artery disease, circulating endothelial progenitor cells, and the role of HMG-CoA reductase inhibitors.. Kidney Int.

[24] Jenuwein T, Allis CD (2001). Translating the histone code.. Science.

[25] Wu X, Li Y, Xue L, Wang L, Yue Y (2010). Multiple histone site epigenetic modifications in nuclear transfer and in vitro fertilized bovine embryos.. Zygote.

[26] Mali P, Chou BK, Yen J, Ye Z, Zou J (2010). Butyrate greatly enhances derivation of human induced pluripotent stem cells by promoting epigenetic remodeling and the expression of pluripotency-associated genes.. Stem Cells.

[27] Bui HT, Wakayama S, Kishigami S, Park KK, Kim JH (2010). Effect of trichostatin A on chromatin remodeling, histone modifications, DNA replication, and transcriptional activity in cloned mouse embryos.. Biol Reprod.

[28] Bird A (1999). DNA methylation.. Science.

[29] Shoemaker R, Wang W, Zhang K (2010). Mediators and dynamics of DNA methylation.. Wiley Interdiscip Rev Syst Biol Med.

[30] Han J, Sachdev PS, Sidhu KS (2010). A combined epigenetic and non-genetic approach for reprogramming human somatic cells.. PLoS One.

[31] Ng HH, Jeppesen P, Bird A (2000). Active repression of methylated genes by the chromosomal protein MBD1.. Mol Cell Biol.

[32] Cheng X, Blumenthal RM (2010). Coordinated chromatin control: structural and functional linkage of DNA and histone methylation.. Biochemistry.

[33] Lyko F (2001). DNA methylation learns to fly.. Trends Genet.

[34] Fry CJ, Peterson CL (2001). Chromatin remodeling enzymes: who's on first?. Curr Biol.

[35] Curradi M, Izzo A, Badaracco G, Landsberger N (2002). Molecular mechanisms of gene silencing mediated by DNA methylation.. Mol Cell Biol.

[36] Si J, Boumber YA, Shu J, Qin T, Ahmed S (2010). Chromatin remodeling is required for gene reactivation after decitabine-mediated DNA hypomethylation.. Cancer Res.

[37] Takahashi K, Tanabe K, Ohnuki M, Narita M, Ichisaka T (2007). Induction of pluripotent stem cells from adult human fibroblasts by defined factors.. Cell.

[38] Hansen JC, Tse C, Wolffe AP (1998). Structure and function of the core histone N-termini: more than meets the eye.. Biochemistry.

[39] Strahl BD, Allis CD (2000). The language of covalent histone modifications.. Nature.

[40] Shi Y, Matson C, Lan F, Iwase S, Baba T (2005). Regulation of LSD1 histone demethylase activity by its associated factors.. Mol Cell.

[41] Lan F, Nottke AC, Shi Y (2008). Mechanisms involved in the regulation of histone lysine demethylases.. Curr Opin Cell Biol.

[42] Huangfu D, Maehr R, Guo W, Eijkelenboom A, Snitow M (2008). Induction of pluripotent stem cells by defined factors is greatly improved by small-molecule compounds.. Nat Biotechnol.

[43] Yamashita T, Kawai H, Tian F, Ohta Y, Abe K (2010). Tumorigenic Development of Induced Pluripotent Stem Cells in Ischemic Mouse Brain..

[44] Ieda M, Fu JD, Delgado-Olguin P, Vedantham V, Hayashi Y (2010). Direct reprogramming of fibroblasts into functional cardiomyocytes by defined factors.. Cell.

[45] Szabo E, Rampalli S, Risueño RM, Schnerch A, Mitchell R (2010). Direct conversion of human fibroblasts to multilineage blood progenitors.. Nature.

